# Driving mitochondrial fission improves cognitive, but not motor deficits in a mouse model of Ataxia of Charlevoix-Saguenay

**DOI:** 10.21203/rs.3.rs-4178088/v1

**Published:** 2024-04-10

**Authors:** Chunling Chen, Ronald A. Merrill, Chian Ju Jong, Stefan Strack

**Affiliations:** University of Iowa, Carver College of Medicine; University of Iowa, Carver College of Medicine; University of Iowa, Carver College of Medicine; University of Iowa, Carver College of Medicine

**Keywords:** ARSACS, ataxia, mitochondrial dynamics, dynamin-related protein 1, protein phosphatase 2A, A kinase anchoring protein

## Abstract

Autosomal-recessive spastic ataxia of Charlevoix-Saguenay (ARSACS) is caused by loss-of-function mutation in the *SACS* gene, which encodes sacsin, a putative HSP70-HSP90 co-chaperone. Previous studies with *Sacs* knock-out (KO) mice and patient-derived fibroblasts suggested that SACSIN mutations inhibit the function of the mitochondrial fission enzyme dynamin-related protein 1 (Drp1). This in turn resulted in mitochondrial hyperfusion and dysfunction. We experimentally tested this hypothesis by genetically manipulating the mitochondrial fission/fusion equilibrium, creating double KO (DKO) mice that also lack positive (PP2A/Bβ2) and negative (PKA/AKAP1) regulators of Drp1. Neither promoting mitochondrial fusion (*B*β2 KO) nor fission (*Akap1* KO) influenced progression of motor symptoms in *Sacs* KO mice. However, our studies identified profound learning and memory deficits in aged *Sacs* KO mice. Moreover, this cognitive impairment was rescued in a gene dose-dependent manner by deletion of the Drp1 inhibitor PKA/Akap1. Our results are inconsistent with mitochondrial dysfunction as a primary pathogenic mechanism in ARSACS. Instead, they imply that promoting mitochondrial fission may be beneficial at later stages of the disease when pathology extends to brain regions subserving learning and memory.

## Introduction

First identified in the Charlevoix-Saguenay region of Quebec, where it was maintained due to founder effects, ARSACS is now recognized as one of the most common recessive spastic ataxia worldwide (1, 2). ARSACS is caused by loss-of-function variants in the *SACS* gene and strikes homozygous carriers in early life. The Sacs knockout (KO) mouse recapitulates many of the cardinal features of ARSACS, including gait abnormalities and loss of cerebellar Purkinje cells (3). Sacsin, the protein encoded by the SACS gene, is a large (~520 kD), multi-domain protein with a predicted function as a HSP70/90 co-chaperone (1).

An early report demonstrated mitochondrial localization of sacsin, as well as an interaction with the mitochondrial fission enzyme Drp1 (4). Mitochondria in neurons with absent or reduced expression of sacsin were abnormally elongated, suggesting that the protein is necessary for proper assembly of the mitochondrial fission machinery. Studies with patient-derived fibroblasts confirmed this conclusion (4, 5). Intermediate filament phenotypes have also been reported, including abnormal bundling of neurofilaments in the soma and dendrites, hypophosphorylation of neurofilament heavy polypeptide (NFH), as well as vimentin cages in patient fibroblasts. In fibroblasts, alterations in the autophagylysosomal system were seen as evidence that sacsin has an important role in proteostasis (3, 6).

With this report, we asked whether dysregulation of Drp1-mediated mitochondrial fission underlies ARSACS pathology, or whether other molecular events, such as intermediate filament aggregation may be primary disease drivers. To this end, we took a genetic approach, crossing *Sacs* KO mice with germline KOs of two well-established regulators of Drp1, PP2A/Bβ2 and PKA/Akap1.

**Figure 1:**
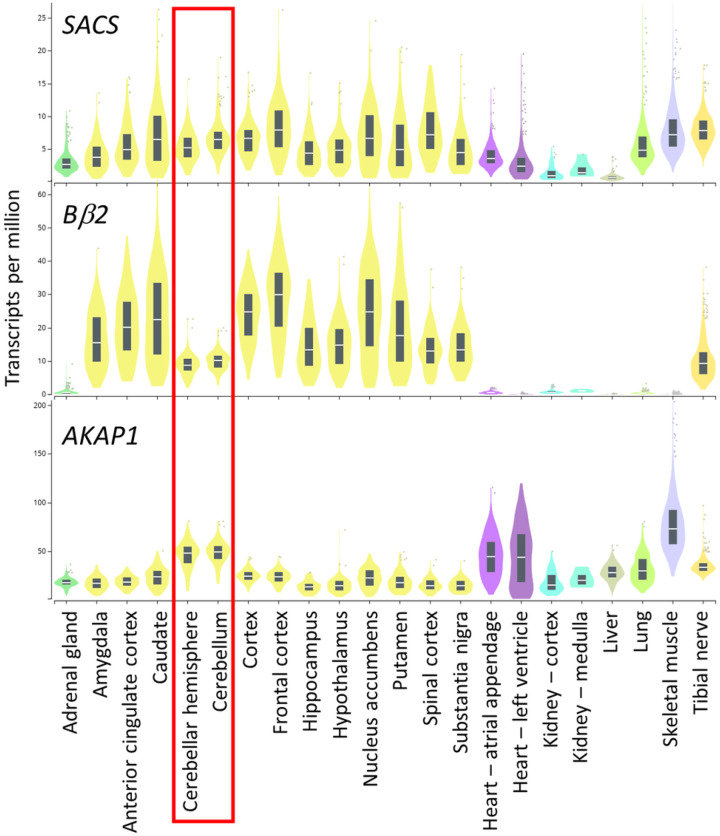
Tissue expression profiles of *SACS*, *AKAP1*, and PP2A/Bβ2 (*PPP2R2B*). Transcript abundance was obtained by human whole-tissue mRNA sequencing and is expressed as transcripts per million. Metadata were procured from the Genotype-Tissue Expression (GTEx) Portal (gtexportal.org) and are displayed with a focus on brain regions.

Mitochondrial fission is regulated by reversible phosphorylation of Drp1 at a highly conserved Ser residue that is phosphorylated by protein kinase A (PKA) and dephosphorylated by two phosphatases, PP2A and PP2B (7). Phosphorylation of Ser637 inhibits Drp1-dependent mitochondrial fission leading to mitochondrial elongation by unopposed fusion, while dephosphorylation activates the fission enzyme, shortening mitochondria (8, 9). We previously reported on mice that lack a regulatory subunit of PP2A (Bβ2), which targets the phosphatase to mitochondria because it includes an alternatively spliced mitochondrial localization sequence (10, 11). Bβ2 is expressed throughout the central and peripheral nervous system, including the cerebellum, but undetectable in non-neuronal cells (Fig. 1). Intriguingly, a non-coding CAG repeat expansion in a promoter region of the gene encoding Bβ2 (*PPP2R2B*) causes spinocerebellar ataxia type-12 (SCA12) (12, 13). In brains of Bβ2 KO mice, Drp1 is hyperphosphorylated at Ser637, and, consistent with Drp1 inactivation, mitochondria are elongated. Bβ2 KO mice are also protected from cerebral ischemic stroke, likely as a consequence of increased bioenergetic reserves (spare respiratory capacity) (11). We further reported that A-kinase anchoring protein 1 (AKAP1) recruits PKA to the outer mitochondrial membrane to phosphorylate and inactivate Drp1 (14). AKAP1 is tethered to the outer-mitochondrial membrane (15) and ubiquitously expressed (Fig. 1). Akap1 KO mice exhibit smaller mitochondria in neurons and glia, along with exacerbated stroke outcomes (16). AKAP1/Bβ2 double KO (DKO) mice show normal Drp1 regulation, mitochondrial morphology, and stroke sensitivity, indicating that PKA and PP2A exert their effects via a shared effector, Drp1 (11).

We generated Sacs/Bβ2 and Sacs/Akap1 DKO mice and tested for ARSACS disease modification at the behavioral level. We reasoned that the Akap1 KO could reverse mitochondrial elongation reported in *Sacs* KO neurons (4). The Bβ2 KO, on the other hand, might attenuate neurodegeneration (as it does in ischemic stroke), if the mitochondrial elongation observed in Sacs KO mice is an adaptive, rather than a disease-driving mechanism. More precipitous cerebellar decline in either Sacs/Bβ2 or Sacs/Akap1 DKO model would also be informative, as it would support the notion of ARSACS as a mitochondrial disease. To our surprise, neither DKO influenced deterioration of motor performance in ARSACS-model mice with age. However, we uncovered a striking decline in cognitive function in older *Sacs* KO mice. Equally striking, this decline was rescued by promoting mitochondrial fission by deleting the Drp1 inhibitor PKA/Akap1.

## Results

Human whole-tissue mRNA sequencing data retrieved from the Broad Institute (gtexportal.org) indicates wide-spread expression of *SACS* and *AKAP1*, whereas Bβ2 (*PPP2R2B*) expression is largely confined to the brain. Within brain regions, *SACS* expression is uniform, while Bβ2 expression is relatively low and *AKAP1* expression is relatively high in the cerebellum (Fig. 1). The three gene products are therefore in the right place to functionally interact.

**Figure 2:**
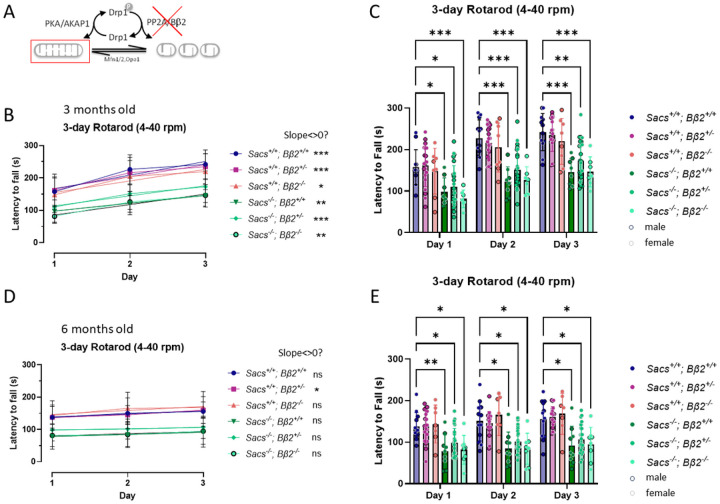
Promoting mitochondrial fusion by deletion of the Drp1 activator PP2A/Bb2 does not improve *Sacs* KO motor performance. (A) Schematic showing that deletion of PP2A/Bβ2 promotes mitochondrial elongation by inhibiting the inhibitory dephosphorylation of the mitochondrial fission enzyme Drp1 at Ser637. (B-E) Motor coordination and motor learning were tested with the accelerating Rotarod test (4–40 rpm over 5 min) at three months (B, C) and six months of age (D, E). Graphs were plotted as the time (second) that mice remained on the rod before falling. (B) At 3 months of age, the linear regression analysis demonstrates significantly different slopes from 0 for all genotypes, indicating motor learning. (C) At the same age, *Sacs*^−/−^ mice performed worse than *Sacs*^+/+^ mice regardless of PP2A/Bβ2 genotype. (D) At 6 months, only SACS^−/−^ Bβ2^+/−^ mice showed motor learning (linear regression slope different from0). (E) At the same age, *Sacs*^−/−^ mice performed worse than *Sacs*^+/+^ mice, again, independent of Bβ2 genotype. Plotted are means ± SD with data points representing individual mice. Black outlines represent male and gray outlines represent female mice. Data were analyzed by linear regression (B, D) or 2-way ANOVA with Dunnett’s post hoc test (C, E); *,p<0.05; **,p<0.01; ***,P<0.001.

We initially examined DKOs of Sacs and the fission driver Bβ2 (Fig. 2A), both of which cause mitochondrial elongation when deleted alone (4, 11). We set up crosses to yield Sacs wild-type and disease-relevant, homozygous null (−/−) mice combined with Bβ2 alleles of all three genotypes (+/+, +/−, −/−). Heterozygous Bβ2 KOs were included because they afford partial protection from ischemic stroke (11). The same cohorts of mice were analyzed at 3 and 6 months of age using the three-day accelerating Rotarod test, which measures motor coordination and motor learning. As reported before (3), *Sacs* deletion by itself significantly impaired performance at both ages. We included both male and female mice in these and subsequent experiments but detected no sex differences. Data from both sexes were therefore pooled for statistical analysis, with data points for male and female mice differentiated by symbol outline colors in each bar graph for transparency.

At three months of age, all six mouse genotypes learned similarly to stay on the accelerating rod (similar slope of day-to-day performance increase), and heterozygous and homozygous deletion of PP2A/Bβ2 did not improve the rotarod performance in Sacs^−/−^ mice (Fig. 2B, 2C). At six months of age, the same cohort of mice did not show a clear learning pattern (Fig 2D). Heterozygous and homozygous deletion of PP2A/Bβ2 did not improve the Rotarod performance in Sacs^−/−^ mice at this age either (Fig 2E).

**Figure 3:**
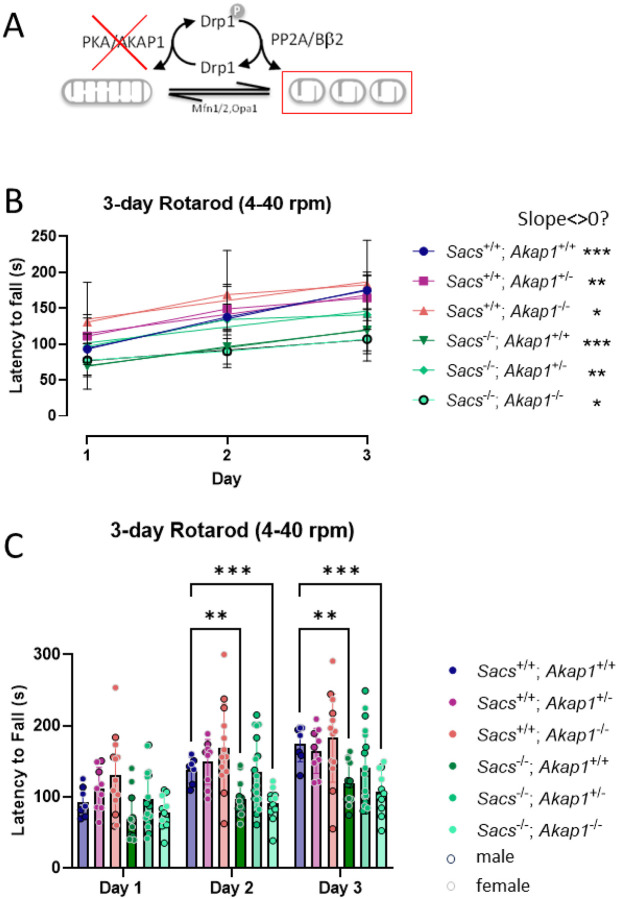
Heterozygous *Akap1* deletion improves motor performance in young *Sacs* KO mice. (A) Schematic showing that deletion of *Akap1* promotes shortening mitochondria by decreasing the inhibitory phosphorylation of the mitochondrial fission enzyme Drp1 at Ser637. (B, C) Motor coordination and motor learning were tested with the accelerating Rotarod test (4–40 rpm over 5 min) at 3 months of age. (B) Regression analysis demonstrates significant motor learning (slopes different from 0) of all genotypes. (C) *Sacs*^−/−^ mice performed consistently worse than Sacs^+/+^ mice, with no effect by homozygous deletion of *Akap1*. However, at day 2 and 3, heterozygous deletion of *Akap1* in Sacs^−/−^ mice performed at a level not significantly different from Sacs^+/+^ mice. See Fig. 2 for data presentation and analysis.

We then investigated Sacs and Akap1 double knockout (DKO) mice to determine if the mitochondrial hyperfusion induced by Sacs knockout (KO) could be reversed by eliminating a restraint on Drp1 fission activity (Fig. 3A). Again, *Sacs*^+/+^ and *Sacs*^−/−^ alleles were paired with all three *Akap1* alleles (+/+, +/−, −/−). Mice with *Akap1*^+/−^ genotype were included because *Akap1* heterozygosity improves neuroanatomical and metabolic symptoms in a mouse model of Bardet-Biedl syndrome (17). 3 months old mice were examined for motor-coordination and -learning using the 3-day accelerating Rotarod test. Motor-performance of mice of all genotypes improved over time, but Sacs^−/−^ mice performed consistently worse than *Sacs*^+/+^ mice (Fig. 3B). Notably, while homozygous *Akap1* deletion did not improve motor function in *Sacs*^−/−^ mice, mice carrying one copy of the *Akap1* gene performed at a level not significantly different from Sacs^+/+^ mice (Fig. 3C).

**Figure 4:**
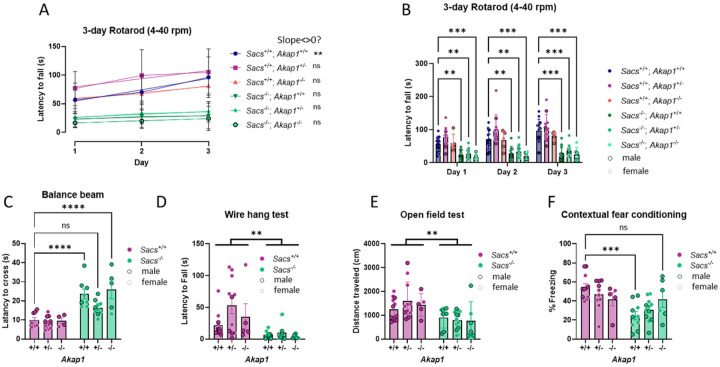
*Akap1* KO rescues cognitive, but not motor function in aged *Sacs*−/− mice. (A, B) According to the 3-day accelerating Rotarod test, aged (13–16 months old) *Sacs*^−/−^ mice performed worse than Sacs^+/+^ mice regardless of *Akap1* genotype. Motor learning was not evident from linear regression analysis. (C) Sacs^−/−^mice took significantly more time to cross the balance beam and homozygous deletion of *Akap1* did not improve their performance. However, *Akap1*^+/−^ mice showed partial improvement. (D) Significant muscle weakness in *Sacs*^−/−^ mice compared to *Sacs*^+/+^ mice was detected by the wire hang test; however, hetero-or homozygous loss of *Akap1* had no effect. (E) In the 10-min open field test, deletion of *Akap1* did not improve the distance-traveled deficit of *Sacs*^−/−^ mice. (F) *Sacs*^−/−^ displayed weaker recall of contextual fear memory. Deletion of *Akap1* improved the learning and memory performance in a gene dose-dependent manner. See Fig. 2 for data presentation and analysis. *Akap1* genotype data in (D, E) was pooled and analyzed by Student’s T-test because there was no significant *Sacs*/*Akap1* genotype interaction.

Encouraged by the finding that Akap1 heterozygosity might alleviate motor deficits, we examined aged (13–16 months old) *Sacs*/*Akap1* DKO mice, when *Sacs* KO symptoms are more pronounced. At this age, Sacs KO mice displayed severe impairments on the Rotarod. However, latency to fall was unaffected by the *Akap1* genotype (Fig. 4A, B). Time crossing the balance beam, an indicator of motor coordination, was increased in *Sacs*^−/−^ mice, with no significant effect of the *Akap1* genotype (Fig. 4C). Distance traveled in the open field test was reduced in *Sacs* KO mice; again, without apparent influence of *Akap1* gene dose (Fig. 4D). Likewise, the wire hang test indicated severely impaired grip strength in *Sacs*^−/−^ mice, but no improvement when one or both *Akap1* alleles had been deleted (Fig. 4E).

Next, the same cohort of aged mice were subjected to an associative learning and memory paradigm, contextual fear conditioning. In this test, mice are placed in a context with novel visual, odor, and tactile cues and then subjected to a foot shock. 24 h later, mice are re-introduced into the same context and the time anticipating the foot shock (“freezing”) is recorded. Compared to *Sacs*^+/+^, *Sacs* ^−/−^ mice displayed a highly significant deficit in associating the context with the foot shock they received the day prior. Remarkably, deletion of Akap1 improved the learning and memory performance in a gene-dose-dependent manner in Sac^−/−^ mice, with homozygous Akap1 deletion resulting in near normal contextual condition (Fig. 4F).

## Discussion

This study confirms a previous report that *Sacs* KO mice faithfully replicate the natural history of ARSACS (3). By analyzing DKOs with established regulators of Drp1, we also provide evidence against dysregulation of the mitochondrial fission enzyme as a primary disease driver in ARSACS. As in other neurodegenerative diseases, mitochondrial dysfunction is increasingly implicated in ARSACS pathology (18–20). For instance, MitoQ, a mitochondria-targeted antioxidant, was recently shown to improve motor coordination and delay Purkinje cell death in *Sacs* KO mice (21). In light of the present results, mitochondrial dysfunction in ARSACS is unlikely due to an imbalance of mitochondrial fission and fusion, but rather a secondary consequence of improper folding and aggregation of one or more of the critical clients of the sacsin co-chaperone complex. In a speculative scenario, aggregated neurofilaments in Sacs KO Purkinje neurons interfere with trafficking of mitochondria along neurites and recycling of dysfunctional mitochondria by mitophagy.

We also report for the first time that loss of sacsin is associated with profound impairments in learning and memory in older mice. Cerebellar ataxias, including ARSACS, manifest not only with motor symptoms, but also with a spectrum of neuropsychiatric and learning disorders including dyslexia, attention deficit hyperactivity disorder, autism spectrum disorders, panic disorder, schizophrenia, and intellectual disabilities. Coined as “cognitive dysmetria” or “cerebellar cognitive affective syndrome”, this was recognized independently in the late 1990’s by Nancy Andreasen (22–24), Jeremy Schmahmann (25), and others (26). Case studies of ARSACS, specifically, listed a variety of non-motor symptoms, such as low motivation (apathy), dysphoria, but also paranoid ideation, irritability, and marked cognitive dysfunction, including anosognosia (27, 28).

Whereas fMRI studies indicate that the cerebellum participates in the retrieval of episodic memory and other cognitive tasks (24), there remain questions how cerebellar disorders impair cognition. On the one hand, cerebellar nuclei project, directly or indirectly, to various brain areas involved in higher-order cognition, including the prefrontal cortex. Also, cerebellar lesions due to accidents or surgical resections can present with non-motor symptoms similar to cerebellar disorders. On the other hand, most cerebellar ataxia disease genes, including SACS, are ubiquitously expressed (Fig. 1), and cerebral atrophy commonly follows cerebellar atrophy in hereditary cerebellar ataxias.

Further studies using conditional Sacs KO mice are needed to pinpoint the cellular and anatomical origins of non-motor symptoms of ARSACS. Also, future studies should address the temporal relationship between cognitive and motor symptoms in ARSACS and the mechanism by which Akap1 deletion improves cognitive decline in Sacs KO mice.

## Materials and methods

### Mice

Mouse work was performed in accordance with the guidelines of the animal ethics committee of the University of Iowa. Mice were group-housed in a colony maintained with a standard 12 h light/dark cycle and given food and water ad libitum. Experiments were performed on age-matched mice of both sexes as indicated in bar graphs. Experiments were conducted according to the Guide for the Care and Use of Laboratory Animals, as adopted by the National Institutes of Health, and with approval of the University of Iowa AAALC-accredited Institutional Animal Care and Use Committee.

The Sacs^−/−^ mice were a kind gift of Bernard Brais, McGill (3). The AKAP1 ^−/−^ mouse line was kindly provided by Dr. Stanley McKnight at University of Washington (29), and Bβ2^−/−^mice were generated at the U. Iowa Mouse KO Core Facility (11). We generated mice with heterozygous or homozygous deletion of PP2A/Bβ2 or *Akap1* that were either wild-type or null at the Sacs locus. Mice were in the C57BL/6J background and were backcrossed to C57BL/6J mice imported from the Jackson Laboratory (Bar Harbor, ME) every 6–10 generations to prevent genetic drift. Mice of all genotypes were born in expected Mendelian ratios and were fertile, except for *Akap1*^−/−^ females who are infertile (29). All mice achieved a normal lifespan, with many individuals surviving beyond two years. However, *Sacs*^−/−^ mice with or without deletion of Bβ2 or Akap1 displayed progressive gait abnormality as documented (3).

### Behavioral Testing

#### General.

5–7 days of habituation and handling was done prior to behavioral assessment. Mice were allowed to acclimatize to the testing environment for 30 minutes prior to the starting of the experiment trials on the day of testing. All genotypes were tested on the same day in a randomized order with experimenters blinded to genotype and sex.

#### Accelerating rotarod.

Mice were placed on a rotating rod (IITC Life Woodland Hills, CA) with gradual speed increase from 4 to 40 rpm over 5 minutes, in which the latency to fall was recorded. Testing consisted of four trials on each day for three consecutive days, with 5–10 minutes between each trial. The average of four daily trials was recorded for each mouse.

#### Balance beam.

The balance beam apparatus was acquired from MazeEngineers Inc (Skokie, IL). The balance beam test was performed as previously described (30). A 12 mm-wide beam was used in our tests. Mice were placed at one end of the beam and the latency for crossing the beam to the other end was recorded by MediaRecorder software (Noldus). Mice were trained for three times in each day for two consecutive days and tested for three times on day 3. The best performance (minimum latency to cross beam) was recorded on the testing day.

#### Wire hang test.

The test began with the mice placed on an elevated wire cage top, which was then inverted and suspended above the home cage. The time it took for the animal to fall was recorded. This test was conducted two times on one day, with 5–10 minutes between each trial. The best performance (longer hang time) was recorded.

#### Contextual Fear Conditioning.

The testing apparatus for 24-hour contextual recall was acquired from CleverSys Inc (Reston, VA). Conditioning was assessed using FreezeScan V.2 software, which measures the “freezing” behavior of the mouse. On the training day, the animals underwent a 3-minute trial, with an electric shock of 1.5 mA delivered at 2.5 min and lasting for two seconds. On the testing day, the animals were returned to the exact same environment and underwent a 5-minute trial without any shocks. Data were presented as percentage of time freezing on the testing day.

#### Statistical Analysis

Data were obtained and analyzed with experimenters blinded to genotype and sex. Statistics were analyzed, and plots were generated using GraphPad Prism software (version 10.2). All data were first analyzed by D’Agostino-Pearson test to determine the normality and then analyzed by two-way ANOVA with Dunnett’s multiple comparison tests. Motor learning was analyzed by linear regression. The false positive rate (a) was set at 0.05.

## Data Availability

Gene expression data (Figure 1) are available from the Genotype-Tissue Expression (GTEx) Portal at gtexportal.org. All other data (Excel spreadsheets, Prism files, movies) can be obtained from the corresponding author upon request.
